# The potassium transporter KdpA affects persister formation by regulating ATP levels in *Mycobacterium marinum*

**DOI:** 10.1080/22221751.2019.1710090

**Published:** 2020-01-08

**Authors:** Xiaofan Liu, Chuan Wang, Bo Yan, Liangdong Lyu, Howard E. Takiff, Qian Gao

**Affiliations:** a Key Laboratory of Medical Molecular Virology (MOE/NHC/CAMS), School of Basic Medical Sciences, Shanghai Medical College and Shanghai Public Health Clinical Center, Fudan University, Shanghai, People’s Republic of China; b Shanghai Public Health Clinical Center, Fudan University, Shanghai, People’s Republic of China; c Integrated Mycobacterial Pathogenomics Unit, Institut Pasteur, Paris, France

**Keywords:** Persister, *Mycobacterium marinum*, potassium, ATP, rifampicin

## Abstract

Mycobacterial persistence mechanisms remain to be fully characterized. Screening a transposon insertion library of *Mycobacterium marinum* identified *kdpA*, whose inactivation reduced the fraction of persisters after exposure to rifampicin. *kdpA* encodes a transmembrane protein that is part of the Kdp-ATPase, an ATP-dependent high-affinity potassium (K^+^) transport system. We found that *kdpA* is induced under low K^+^ conditions and is required for pH homeostasis and growth in media with low concentrations of K^+^. The inactivation of the Kdp system in a *kdpA* insertion mutant caused hyperpolarization of the cross-membrane potential, increased proton motive force (PMF) and elevated levels of intracellular ATP. The KdpA mutant phenotype could be complemented with a functional *kdpA* gene or supplementation with high K^+^ concentrations. Taken together, our results suggest that the Kdp system is required for ATP homeostasis and persister formation. The results also confirm that ATP-mediated regulation of persister formation is a general mechanism in bacteria, and suggest that K^+^ transporters could play a role in the regulation of ATP levels and persistence. These findings could have implications for the development of new drugs that could either target persisters or reduce their presence.

## Introduction

Tuberculosis (TB), caused by *Mycobacterium tuberculosis*, kills more people than any other single infectious disease*.* Drug regimens can cure 90–95% of patients with drug susceptible TB, but only if the drugs are taken for at least six months [1]. One factor thought to contribute to the need for prolonged therapy is the presence of “persisters” – bacilli that are not rapidly killed by the antibiotics currently used [2]. Persistence is a ubiquitous phenomenon in bacteria that greatly hinders the effectiveness of anti-bacterial treatments [3], and survival of the persisters in the presence of antibiotics may increase the likelihood of acquiring resistance mutations [4]. Therefore, studying the mechanism of mycobacterial persisters could lead to the development of new drugs that can effectively eliminate them, thereby both shortening the duration of TB chemotherapy and reducing the emergence of drug-resistant *M. tuberculosis* strains [5].

Drug resistance develops through mechanisms that prevent the drugs from interacting with their targets. There are many ways this can be accomplished [6] including, among others, mutations in the drug targets, mutations that compromise the enzymes required to activate prodrugs, and regulatory mutations that increase the expression of either enzymes that inactivate the drugs or efflux pumps that remove the drugs from the bacteria [7,8]. In contrast, persistence is the ability to survive antibiotic treatment without acquiring resistance mutations [9].

Persistence is believed to be a transient dormant state, and the proportion of persisters in the population varies with the environment, making the study of persistence quite challenging [3]. The mechanisms controlling persistence are not fully understood, but previous studies have found that persistence in bacteria is associated with toxin anti-toxin genes, the SOS response, the DNA repair system, energy metabolism, the stress response, phosphate metabolism and other processes [10–21]. In *M. tuberculosis*, genes encoding isocitrate lyase [22], the triacylglycerol metabolism-related protein Tgs1 [23], and the phosphate metabolism regulator PhoY2 [24–26] have all been implicated in the formation of mycobacterial persisters, suggesting that bacterial growth and metabolism are closely related to persister formation.

K^+^ is essential for many cellular functions, including the maintenance of intracellular pH and the cross-membrane potential (Δ Ψ) [27–30]. During the course of infection and disease, *M. tuberculosis* must sense and adapt to the different K^+^ concentrations encountered in intracellular and extracellular environments. Mycobacteria regulate K^+^ transport with the Trk and Kdp systems. The Trk system is thought to be the principal K^+^ transport system, and an *M. smegmatis* mutant with a *trkA* mutation showed an increased cross-membrane potential that was associated with altered antibiotic susceptibilities [28]. In addition, the growth of the *trkA* mutant was severely impaired in mildly acidic conditions [31,32] unless supplemental K^+^ was added to the growth medium. The Kdp system is inducible and encoded by *kdpFABC* and *kdpDE*. This system becomes operational under low K^+^ concentrations (∼2 μM) when other K^+^ uptake systems such as Trk fail to function [33].

*M. marinum,* a close relative of *M. tuberculosis*, has been used previously to study mycobacterial persistence [34–36]. Here we report that a screen for genes associated with the persistence of *M. marinum* in the presence of antibiotics yielded *kdpA*, which is predicted to encode a high-affinity K^+^ transporter that influences growth under low K^+^ conditions. A *kdpA* mutant showed a decrease in the *M. marinum* persistence ratio, but persister formation could be restored by increasing the K^+^ concentration. In addition, the *kdpA* mutant strain had increased membrane potential and high ATP levels. These findings confirm other reports [37] that persister formation is correlated with ATP levels, and suggest that the ATP levels could be regulated by K^+^ transport.

## Materials and methods

### Bacterial strains, medium, and growth conditions

*M*. *marinum* M (ATCC BAA-535) was used as the wild type strain in this study. *M*. *marinum* strains were grown at 32°C in Middlebrook 7H9 broth or on 7H10 agar enriched with 10% oleic acid-albumin-dextrose-catalase (OADC), and 0.4% volume/volume (v/v) glycerol. To reduce bacterial clumping, 0.02% v/v tyloxapol (Ty) was also added to the 7H9 broth (Middlebrook 7H9 OADC-Ty). When indicated, 25 μg/mL kanamycin, 25 μg/mL gentamycin and 50 μg/mL hygromycin were added to the growth medium. For mutant selection, medium was supplemented with 25 μg/mL kanamycin or 50 μg/mL hygromycin. For K^+^ limitation studies, the *M. marinum* was cultured in 7H9 broth in which KH_2_PO_4_ was replaced by NaH_2_PO_4_. For K^+^ enrichment studies, KCl was used to increase K^+^. For pH studies, the phosphate buffer NaH_2_PO_4_/Na_2_HPO_4_) was used to adjust the pH to 5.5. *Escherichia coli* DH5a was cultured at 37°C in Luria–Bertani (LB) medium containing 50 μg/mL kanamycin or 150 μg/mL hygromycin, as appropriate.

### Screening an *M. marinum* φMycoMar T7 transposon insertion library

Propagation of the φMycoMar transposon phage and preparation of phage lysates have been described previously [38]. The *M. marinum* transposon insertion library was spread onto Middlebrook 7H10-OADC plates supplemented with kanamycin and incubated at 32°C. A total of 9216 colonies were picked randomly, restreaked and inoculated into individual wells of 96-well microtiter plates containing Middlebrook 7H9 OADC-Ty. To screen for growth defect mutants in the presence of low concentration of rifampicin, the colonies were inoculated into Middlebrook 7H9 OADC-Ty and Middlebrook 7H9 OADC-Ty supplemented with rifampicin at 0.125 μg/mL. Those that did not grow or grew slowly in media with rifampicin were streaked onto Middlebrook 7H10-OADC plates containing rifampicin (0.125 μg/mL). Strains that showed sensitivity to the drug were retested twice and those that were confirmed to have growth defects in the presence of 0.125 μg/mL rifampicin were used for further study.

Localization of the transposon insertions was determined as previously described [38]. Briefly, total chromosomal DNA was cleaved by *BamHI*, then self-ligated with the T4 DNA ligase and transformed into E. coli DH5a l pir116. Plasmid DNA was isolated from the Km^R^ *E. coli* transformants. MycoMar-specific primers were used to determine the DNA sequence at the transposon/chromosomal junction, and these DNA sequences were compared with the genome sequence of *M. marinum*.

### Complementation of mutant strains

A DNA fragment containing the *kdpA* gene was obtained by PCR amplification of *M*. *marinum* genomic DNA using forward primer 5′-ATACTAGTTAGGTGAGTACCACGATGGC and reverse primer 5′-ATAAGCTTCGACAGTGGTGCTCATCAG. The amplified fragment was digested with *SpeI* and *HindIII* and cloned into the pSMT3L × EGFP vector [39]. The pSMT3L-(*kdpA*) plasmid was electroporated into the *kdpA* mutant stains and transformants were selected on Middlebrook 7H10-OADC plates containing kanamycin and hygromycin.

### Determination of minimal inhibit concentration (MIC) and formation of persisters

Antibiotic MIC’s were determined with serial 2-fold dilutions in Middlebrook 7H9 OADC-Ty. The MIC was recorded as the minimum drug concentration that prevented visible growth.

For drug exposure persister assays, the strains were grown in Middlebrook 7H9 OADC-Ty to exponential (OD600 = 1.0) or stationary growth phase (4 days after OD600 = 2.0) and then rifampicin was added to a final concentration of 25 μg/mL. The drug exposure was carried out over a period of 3 days at 32°C with shaking (120 rpm). Aliquots of bacterial cultures exposed to the drug were taken at different timepoints and washed in PBS buffer before plating for survivors on Middlebrook 7H10-OADC plates. The CFU was calculated by plating 100 μL from each of three 10 - fold serial dilutions for each culture, with three plates for each dilution. The number of surviving colonies after antibiotic exposure was counted after 7 days of growth at 32°C, and the surviving fraction was calculated as the ratio of surviving colonies to total CFU present in the cultures, as determined from serial dilutions of the culture just before exposure to rifampicin.

### Membrane potential and intracellular ATP production assays

The transmembrane potential was determined with the MitoProbe™ DiOC_2_(3) Assay Kit (Invitrogen™), and the cellular ATP levels were measured using a firefly luciferase-based ATP assay kit (Beyotime, Shanghai, China), according to the manufacturers’ protocols.

### Quantitative reverse-transcriptase PCR (qRT-PCR)

The transcriptional expression of selected genes was measured by qRT-PCR using the primers listed in Table S4, with *sigA* as an internal control. The cDNA was prepared from RNA isolated from each of three independent cultures of each strain. The qRT-PCR reactions were carried out using SYBR Premix Ex Taq II (2×) (TAKARA) and a CFX96™ Real-Time PCR System (Bio-Rad).

### Statistical analysis

The CFU data were analysed for statistical significance using one-way ANOVA. The growth curves were analysed for statistical significance with RM one-way ANOVA. ATP levels and membrane potentials were analysed for statistical significance using paired *t*-tests.

## Results

### Identification of kdpA as a new persister gene

We previously observed that strains lacking the persistence-related gene *phoY2*, showed poor growth in medium with sub-MIC antibiotic concentrations [25]. To identify other persistence-related genes, we screened a library of transposon insertion mutants for growth after 3–5 days in liquid media with 0.125 ug/mL RIF, which is half the RIF MIC for *M. marinum*. A total of 18 mutant strains were confirmed to have a growth defect in liquid media with 0.125 μg/mL rifampicin, and the transposon insertion sites of 15 of these mutant strains were identified (Table S1). Some of the mutants, (e.g. *mas*::Tn and *fstW*::Tn, Table S2) showed no growth, and thus had a lower MIC than the wild type strain. Others, such as the *kdpA*::Tn mutant, grew slower than the wild type strain in 0.125 ug/mL RIF, but the RIF MIC was the same as for the wild type strain (Table S2).

**Figure 1.**
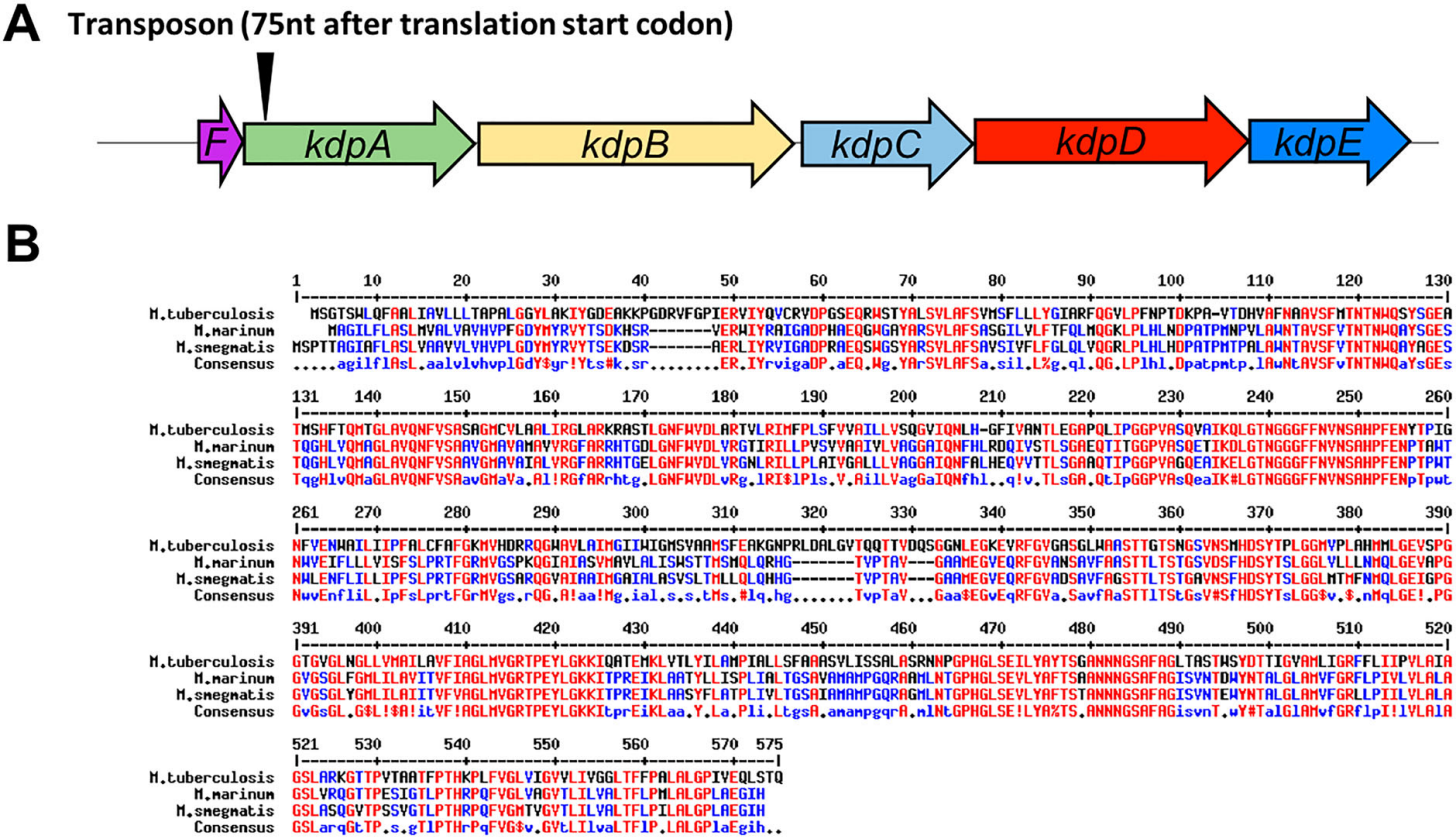
Analysis of the *M. marinum kdpA* gene. (a) The arrowhead indicates the location of the transposon insertion in the rifampicin-sensitive mutant. (b) Alignment of *kdpA* sequences from *M. marinum* (MMAR_0631), *M. smegmatis* (MSMEG_5392), *M. tuberculosis* (Rv1029), and *E. coli* (MG1655). Red represents high consensus, blue represents low consensus, black represents neutral.

In one of these selected mutants, the transposon was inserted 75 nt after the translation start codon of gene *MMAR_0631* (Figure 1(a)), which encodes KdpA, part of the Kdp-ATPase system. The Kdp-ATPase system is a K^+^ uptake mechanism that is widely distributed in different bacterial and archeal genera [40–43], and has been found in many mycobacteria, including *M. tuberculosis, M. avium, M. bovis, M. smegmatis* and *M. marinum* [29,44]. The *M. marinum* KdpA protein shares high sequence identity with the KdpA protein in *M. smegmatis*, suggesting that the function of KdpA may be similar (Figure 1(b)). The Kdp-ATPase system is composed of several subunits: KdpA is involved in binding and translocating K^+^; the KdpF is required for stabilizing the complex; and the association of KdpB with KdpC is essential for ATP hydrolysis [45] which provides the energy for the K^+^ uptake. The system is usually regulated by the KdpD / KdpE two-component system.

### Decreased persisters in kdpA mutant upon rifampicin exposure

**Figure 2.**
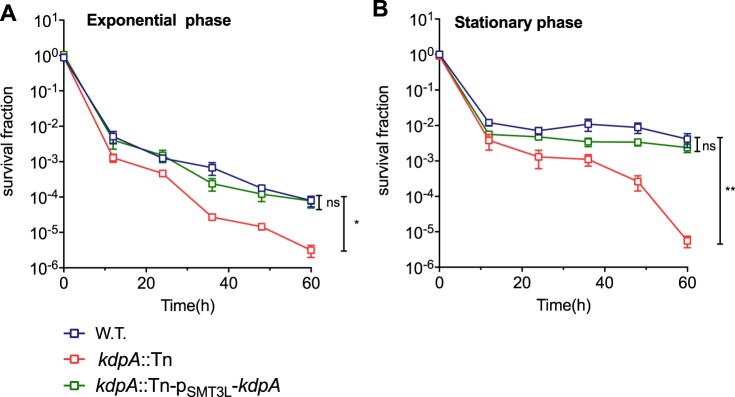
Susceptibility of the *kdpA* mutant to antibiotics. Survival fraction of the wild type (W.T.), the *kdpA* mutant (*kdpA*::Tn), and the complemented strain (*kdpA*::Tn-p_SMT3L_-*kdpA*) after rifampicin exposure. The wild type, *kdpA* mutant, and complemented strains were exposed to 25 μg/mL rifampicin in (a) the exponential growth phase or (b) the stationary growth phase, and survival was determined by spot plating. Colonies were counted after 7 days of growth. The graphs show the average of at least three independent experiments. Error bars indicate Std.Dev. One-way ANOVA was used for the statistical analysis: ns indicate *p* > .05, *indicates *p* < .05, **indicates *p* < .01.

Persisters are characterized by their tolerance to different antibiotics [46], and an *M. smegmatis* mutated in the Trk K^+^ transporting system was found to have altered MICs for several antibiotics, including rifampicin and isoniazid [28]. We compared the *kdpA* mutant with the wild type strain, and found no differences in the MICs for isoniazid, streptomycin, gentamicin, ciprofloxacin, or rifampicin (Table S2) [16,47]. In a killing curve experiment however, the *kdpA* mutant showed decreased persister formation. After 60 h exposure to rifampicin, the survival fraction of the *kdpA* mutant was lower than for the wild type (Figure 2(a,b)), but complementation of the *kdpA* mutant with plasmid pSMT3L-(*kdpA*) restored the persister formation to the wild type level. With bacteria from exponential phase cultures, the survival fraction of the *kdpA* mutant was 25-fold lower than in the wild type strain, but when stationary phase cultures were exposed to rifampicin, the survival fraction of the *kdpA* mutant was about 735-fold less than obtained with the wild type or complemented strains.

### The Kdp-ATPase system is a K^+^ uptake system and the growth rate in the kdpA mutant is altered under low K^+^ conditions

**Figure 3.**
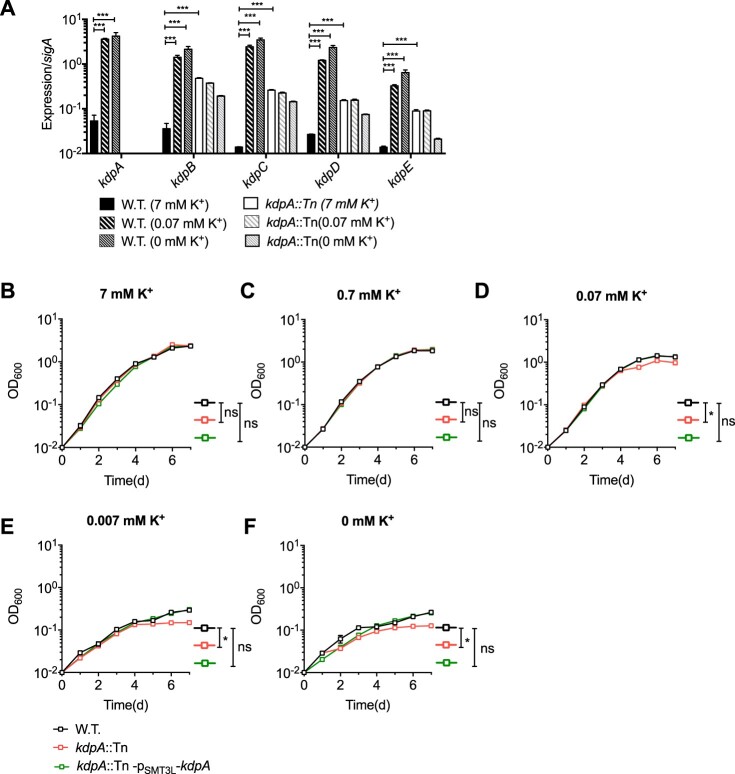
Expression of the *kdp* operon and its effect on growth in varying K^+^ concentrations. (a) Relative expression levels of the *kdp* genes during growth at 7–0.07 mM K^+^. RNA was extracted when the bacteria grew to OD_600_ = 1. For strains grown in 0.007 mM or 0 mM K^+^, RNA was extracted 4 days after inoculation. Total RNA was extracted and cDNA was used for quantitative analysis. Data were analysed with the relative quantification (ddCt) method, using the *sigA* gene as a reference for normalization. The graphs show the average of three biological replicates. Error bars indicate Std.Dev. (b–f) Growth curves for the wild type, *kdpA* mutant, and complemented strains in medium with: (b), 7 mM K^+^; (c), 0.7 mM K^+^; (d), 0.07 mM K^+^; (e), 0.007 mM K^+^ or (f), no K^+^. One-Way ANOVA was used for the statistical analysis: ns indicate *p *> .05,*indicates *p* < .05; and ***indicates *p* < .001.

In other bacteria, such as *E. coli*, *M. smegmatis* and *Staphylococcus aureus* [33,48,49], the Kdp-ATPase is a high affinity inducible system that is important for survival in environments with low K^+^ concentrations [33]. Therefore, we next assessed the relative expression levels of *kdpABC* and *kdpDE* by qRT-PCR in strains grown in the normal growth medium or medium with low K^+^ concentrations. In the wild type strain, the expression of the *kdp* genes was very low in growth medium with the routine 7 mM K^+^ concentration, but expression greatly increased when the concentration of K^+^ was 0.07 mM or lower (Figure 3(a)). In the *kdpA* mutant, however, the expression of *kdpBC* and *kdpDE* was low in normal growth medium and did not increase when the K^+^ concentration was reduced (Figure 3(a)). This indicates that *kdpABC* and *kdpDE* expression is normally induced under K^+^-limiting conditions, but the induction does not occur in the mutant with a transposon insertion in *kdpA*.

To further examine the role of KdpA in K^+^ transport, we tested the growth of the *kdpA* mutant at various K^+^ concentrations (Figure 3(b–f)). In medium with 7 mM or 0.7 mM K^+^, there was no significant growth difference between the wild type strain and the *kdpA* mutant, but in 0.07 mM K^+^ or lower the mutant strain grew more slowly than the wild type. This suggests that, as in other bacteria, the KdpA system in *M. marinum* is a high K^+^ affinity transport system. The *kdpA* insertion abrogated the induction of the Kdp system in K^+^-limited conditions, presumably resulting in reduced K^+^ uptake and consequently a significant growth defect in environments with low K^+^ concentrations.

### KdpA is required for pH homeostasis in *M. marinum*

**Figure 4.**
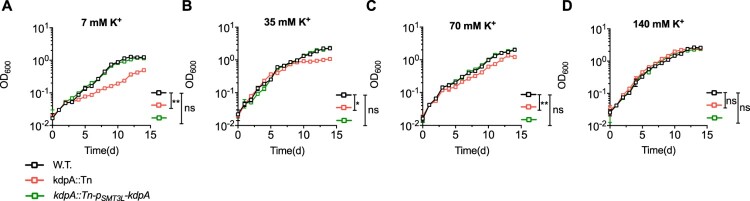
The effect of K^+^ concentration and extracellular pH on the growth of the wild type, *kdpA* mutant, and complemented strains. Growth in medium at pH 5.5 containing: (a), 7 mM K^+^; (b), 35 mM K^+^; (c), 70 mM K^+^; and (d), 140 mM K^+^. KCl was used to increase K^+^ and OADC was not added to the medium. The figures show the average of three biological replicates. Error bars indicate Std.Dev. One-Way ANOVA was used for the statistical analysis: ns indicate *p *> 0.05, *indicates *p* < .05; and **indicates *p* < .01.

Because K^+^ accumulation is essential for maintaining intracellular pH homeostasis [28,50], the disruption of K^+^ uptake could increase susceptibility to acidic conditions. We tested the role of KdpA in maintaining pH homeostasis by culturing *M. marinum* in medium at pH 5.5 with a range of K^+^ concentrations (Figure 4). In acidic medium with 7 mM K^+^, the growth of the wild type and the complemented strains was impaired but significantly better than the growth of the *kdpA* mutant (Figure 4(a)). The growth of all strains improved with 35 and 70 mM K^+^, but the growth of the *kdpA* mutant was still significantly worse. With 140 mM K^+^, however, the growth of the *kdpA* mutant was nearly identical to that of the wild type and complemented bacteria, showing no apparent growth impairment (Figure 4(d)). These results show that the KdpA is needed for pH homeostasis when cells are exposed to an acidic environment in low or moderate K^+^ concentrations, but is not required in the presence of abundant K^+^.

### Supplementation with K^+^ restored persister formation of rifampicin in the kdpA mutant

**Figure 5.**
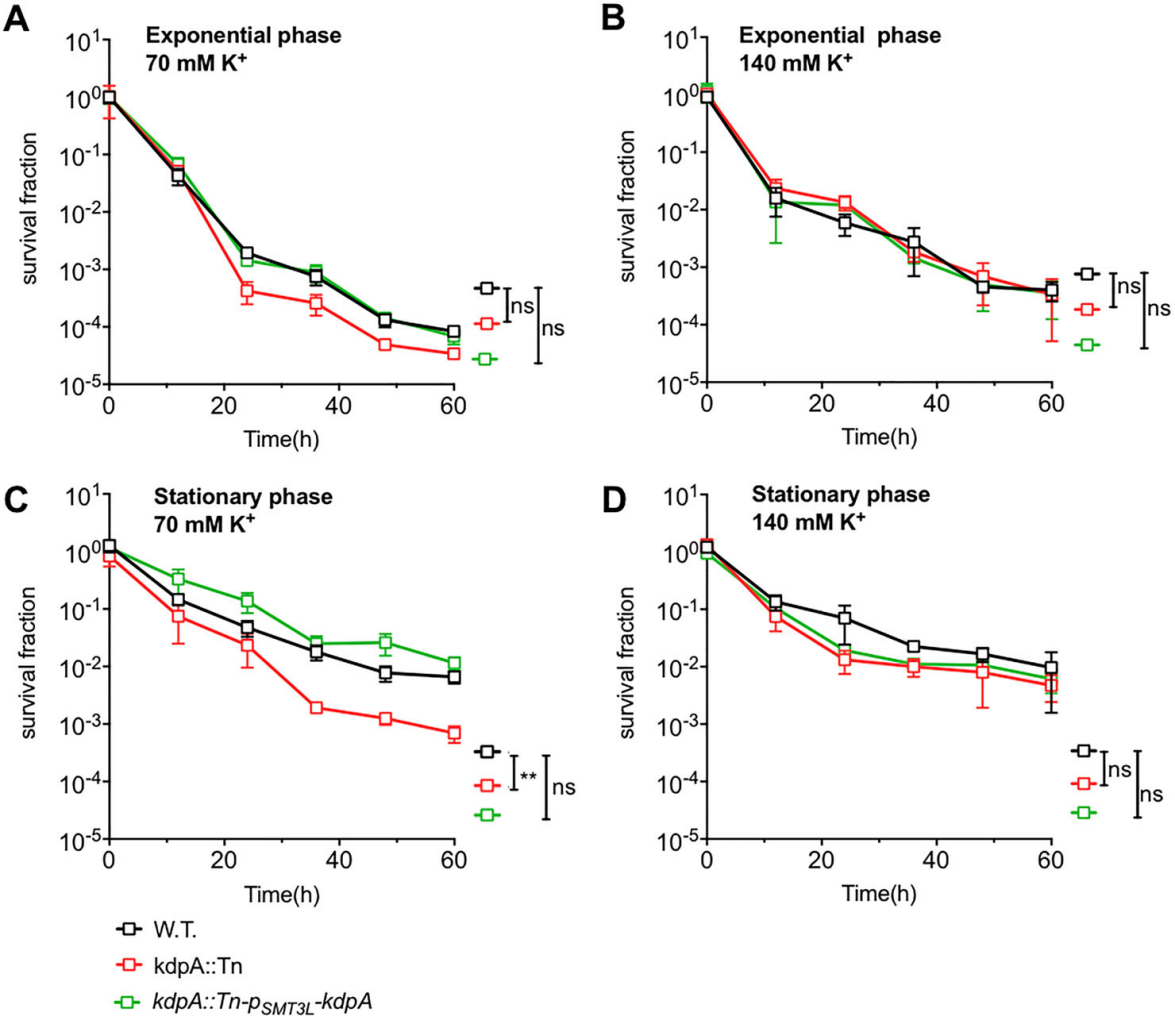
Survival fraction of the wild type, *kdpA* mutant, and complemented strains after exposure to 25 μg/mL rifampicin in 70 Mm or 140 mM K^+^. The strains were exposed to rifampicin in the exponential growth phase (a and b) or the stationary growth phase (c and d). Survival was determined by spot plating of aliquots taken after various times of growth in rifampicin. Colonies were counted after 7 days of growth. KCl was used to increase K^+^. The graphs show the average of three biological replicates. Error bars indicate Std. Dev. One-way ANOVA was used for the statistical analysis: ns indicate *p *> .05, *indicates *p* < .05, **indicates *p* < .01.

As the mutation of *kdpA* led to a decline in persister formation, and *kdpA* is highly expressed by *M. marinum* under low K^+^ conditions when it is required for growth, we speculated that K^+^ may play an important role in persistence. To explore the role of K^+^ in persister formation, we compared the proportion of persisters after rifampicin exposure (25 ug/mL) in the presence of a range of K^+^ concentrations. In medium with 7 mM K^+^, the *kdpA* mutant exhibited markedly decreased persister formation (Figure 2(a,b)). With 70 mM K^+^, the survival fraction of the *kdpA* mutant taken from exponential phase cultures was no statistical difference compared with the wild type strain (Figure 5(a)), but when stationary phase cultures were used, the survival fraction of the *kdpA* mutant was approximately 9.5-fold lower (Figure 5(c)). When the concentration was increased to 140 mM K^+^, the survival fractions of the *kdpA* mutant from both exponential and stationary phase cultures were nearly identical to those of wild type and complemented strains (Figure 5(b,d)). These data indicate that similar to the involvement of KdpA in maintaining pH homeostasis, an intact KdpA-dependent K^+^ transport system is required for the formation of persisters in low or moderate K^+^ concentrations, but is not needed in high concentrations of K^+^.

### KdpA mutation leads to hyperpolarization of membrane potentials and increased intracellular ATP levels

The cross-membrane potential, ΔΨ, is the electrochemical potential created when the inside of the bacterial is more negatively charged than the outside, and previous work has shown that K^+^ uptake has a direct influence on ΔΨ [28,51]. To investigate whether the *kdpA* mutant affected the cross-membrane potential, we first analysed the effect of carbonyl cyanide m-chlorophenyl hydrazone (CCCP), an H^+^ ionophore that permits the unregulated entry of H^+^ into the bacteria. When the K^+^ concentration was 7 mM, the *kdpA* mutant was more tolerant to CCCP, with an MIC that was 3-fold higher than that of the wild type or complemented strains, but the difference narrowed when the K^+^ concentration was 70 mM or 140 mM (Table S3).

**Figure 6.**
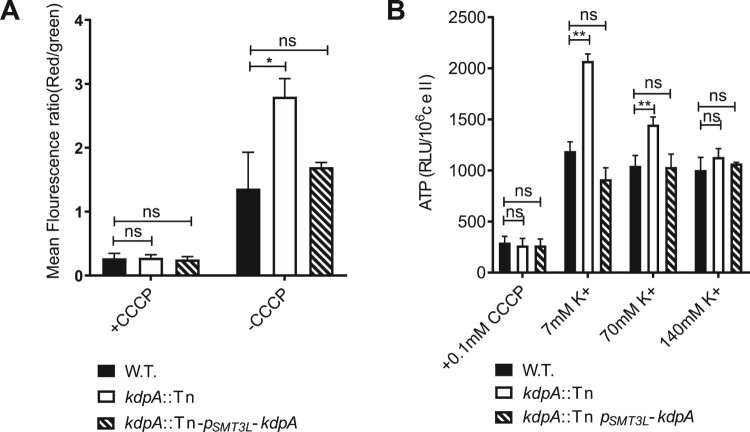
*kdpA* mutant cells have a higher membrane potential and intracellular ATP level than wild type cells. (a).The mean fluorescence intensity ratio (red:green) of the wild type, *kdpA* mutant, and complemented strains, incubated with DiOC_2_(3) for 15 min in either the presence or absence of CCCP. (b). ATP levels were measured in 7 mM K^+^, 70 mM K^+^, and 140 mM K^+^. The graphs show the average of three biological replicates. Error bars = Std.Dev. The differences between the wild type, *kdpA* mutant, and complemented strains were analysed by multiple *t*-tests: ns indicates *p* > .05; *indicates *p* < .05; **indicates *p* < .01.

To assess the effect of the loss of the Kdp system on the ΔΨ, we used the cationic fluorescent dye DiOC_2_(3), which becomes more red as the cross-membrane potential increases. As shown in Figure 6(a), the *kdpA* mutant stained with DiOC_2_(3) exhibited a higher red:green fluorescence ratio than the wild type or complemented strains, demonstrating an increased, hyperpolarized ΔΨ. In the presence of CCCP, the ΔΨ was dissipated in all three strains.

The Proton Motive Force (PMF) is enabled by a higher proton concentration outside than inside the bacteria, and the ATP synthase F_0_F_1_ utilizes the PMF to drive ATP production [52,53]. Because the inactivation of the Kdp system in the *kdpA* mutant caused cross-membrane hyperpolarization, we hypothesized that the intracellular ATP concentration would also be increased, and used a luciferase-based assay to quantify the intracellular ATP concentrations. In medium with a routine K^+^ concentration of 7 mM, the *kdpA* mutant had a 1.67-fold increase in cellular ATP compared with the wild type strain (Figure 6(b)), but in the presence of abundant K^+^, 140 mM, the difference disappeared.

## Discussion

The survival of *M. tuberculosis* persisters in the presence of antibiotics is thought to be one of the principle reasons why anti-tuberculosis therapy must be given for at least six months to obtain 90–95% cure rates [1,2]. To learn more about the mechanisms responsible for persistence in mycobacteria, we screened an *M. marinum* library of transposon insertions to find mutants with an impaired ability to form persisters in the presence of rifampicin. One of the mutants obtained had an insertion in *kdpA*, the second gene in an apparent operon whose encoded proteins constitute the Kdp K^+^ transport system (Figure 1). The Kdp system, which is widely conserved in bacteria and archaea, is a high-affinity K^+^ transport system that functions to maintain K^+^ homeostasis in low K^+^ conditions. The transposon insertion into *kdpA* completely inactivates this transport system because *kdpA* encodes the membrane channel subunit, and the insertion near the start of the *KdpFABCDE* operon apparently prevents its induced expression in low K^+^ conditions (Figure 3). Our results demonstrate that the Kdp system is required for growth and maintenance of intracellular pH homeostasis in low K^+^ conditions, and its absence results in hyperpolarization of the membrane, increased PMF and higher levels of intracellular ATP, which likely explains its involvement in the formation of persisters. We also found two additional mutants, one with a transposon insertion into KdpD and one with an insertion into KdpE. Both mutants were incapable of inducing expression of the *Kdp* genes in medium with low K^+^ concentrations (Figure S1). These two genes encode the two-component regulatory system involved in the regulation of the expression of the Kdp system in other bacteria, and apparently have a similar role in *M. marinum*.

There is a growing body of evidence that dormancy and persistence are associated with low levels of intracellular ATP. In *S. aureus*, ATP levels were significantly reduced in stationary phase, and when the high ATP levels in the exponential phase were reduced by treatment with arsenate, there was a 325-fold increase of persister formation [37]. In contrast, when the culture medium was supplemented with glucose to increase metabolism, the intracellular ATP levels increased and there was a 100-fold reduction in the proportion of persisters [37].

Dormant bacteria have low levels of ATP and increased drug tolerance, associated with lower metabolic activity and reduced cytoplasmic fluidity [54] that has been related to protein aggregation [55]. The increase in ATP levels that accompanies the transition from dormancy reduces protein aggregation and increases the cytoplasmic fluidity [16,56–58], thereby facilitating metabolic processes, many of which require the energy of ATP [54,56]. ATP is produced by the F_0_F_1_ ATP synthase that is driven by the PMF [52,53]. Therefore, anything that decreases the PMF will reduce ATP production and favour dormancy and persistence, and conversely, anything that increases the PMF will increase ATP production and decrease dormancy and persistence. In this study, the absence of the Kdp system reduced K^+^ uptake, especially in low K^+^ conditions, and the resultant increase in the intracellular negative charge and the PMF led to an increase in the production of ATP and a decrease in the formation of persisters.

This finding is consistent with mechanisms that have been described for some toxin/antitoxin systems that increase the formation of persisters. TisB is part of an endogenous type I toxin–antitoxin system in *E. coli* whose expression leads to an increase in persister formation. The small TisB peptide localizes to the inner bacterial membrane, where it forms a channel allowing a cation influx that reduces the PMF, thereby resulting in lower levels of ATP [16]. A very similar effect has been attributed to the toxin GhoT, which belongs to the type V toxin/antitoxin system in *E. coli* [16,57,59]. Like TisB, GhoT also damages the membrane, which leads to a loss of the PMF, lower levels of ATP and reduced metabolism, which is associated with increased dormancy and persistence in the presence of various antibiotics [57].

A previous study of an *M. smegmatis* mutant with a deletion of the TrkA system, the main K^+^ transporter, also found defects in growth and pH homeostasis, along with membrane hyperpolarization at normal K^+^ concentrations [28]. The formation of persisters in the *M. smegmatis trkA* mutant was not assessed, and the intracellular ATP levels were not measured. The *M. smegmatis trkA* mutant had reduced MICs for isoniazid and aminoglycosides, perhaps because the higher negative charge of the intracellular membrane facilitated entry of these positive charged antibiotics. The *trkA M. smegmatis* mutant also had increased MICs for rifampicin and novobiocin, and disruption of the TrkA system in *E. coli* increased the MICs for doxycycline, tetracycline and nalidixic acid [60]. One possible explanation is that the increased PMF in the Trk mutants led to increased activity of proton antiporter efflux transporters [61] that pumped these antibiotics out of the bacteria. Antibiotic specificity similar to that of *trkA* was also found in the *kdpA* mutant, where we found reduced persistence to streptomycin (Figure S2) but no change in isoniazid (Data not show). The mechanisms affected by *kdpA* that are involved in the generation of persister cells are specific for particular antibiotics, which has also been previously reported [62].

Persisters appear to be bacteria within a growing culture that are in a stationary-like dormant state [63]. While ATP depletion may be the principal mechanism of persister formation [63], it remains a mystery why normally only a small fraction of bacteria in a growing culture develop low levels of ATP and enter into the dormant, persister state. Is there a specific mechanism governing the formation of persisters, or simply a stochastic variation in the expression of some particular genes? Here, we discovered a new aspect of basic *M. marinum* physiology that is associated with persister formation*.* The loss of function of a K^+^ transport channel protein, KdpA, led to an increase in the PMF, higher levels of intracellular ATP and a decrease in persister formation. Presumably, an increase in K^+^ transport could lead to a decreased PMF, lower levels of ATP and consequently a dormant or persister state.

Because the Kdp system uses its ATPase activity to import K^+^, it seems feasible that the Kdp system could be involved in PMF homeostasis or regulation of the ATP levels, and perhaps also the transition out of a dormant or persister state. In this scenario, the decreased ATP levels in dormancy would reduce Kdp mediated K^+^ influx, leading to an increased PMF that would drive the F_1_F_0_ ATP synthetase to increase ATP levels, which would then cause the bacteria to exit the dormant state. While this regulatory role for the K^+^ transport systems has yet to be demonstrated, our results suggest that K^+^ flux is another aspect of bacterial biology that could be involved in dormancy and persistence.

## Supplementary Material

Supplemental Material
