# Supplementary material for: The potassium transporter KdpA affects persister formation by regulating ATP levels in Mycobacterium marinum
Source: Emerg Microbes Infect. 2020 Jan 8;9(1):129–39. doi: 10.1080/22221751.2019.1710090 (PMC6968386; doi:10.1080/22221751.2019.1710090)
Supplement: Supplemental Material [file TEMI_A_1710090_SM6276.docx]

**Supplemental Figure 1**: Relative expression levels of the *kdpABC* and *kdpDE* genes of *kdpD* and *kdpE* mutants in varying K^+^ concentrations. Total RNA was extracted and cDNA was used for quantitative analysis, with the expression of *sigA* as a reference for determining the relative expression levels. The graphs show the average of three biological replicates. Error bars indicate Std.Dev. The differences were analyzed by multiple t tests: ns indicate p＞0.05; *** indicates p < 0.001.

**Supplemental Figure 2**. Survival fraction of the wild type (W.T.), the *kdpA* mutant (*kdpA*::Tn), and the complemented strain(*kdpA*::Tn-*pSMT3L*-*kdpA*) after streptomycin exposure. The three strains were exposed to 250 μg/mL streptomycin in 7 mM K^+^ (A and B), or 140 mM K^+^ (C and D), and survival was determined by spot plating. The colonies were counted after 7 days of growth. The graphs show the average of at least three independent experiments. Error bars indicate Std.Dev. One-Way ANOVA was used for the statistical analysis: ns indicate p＞0.05 ,* indicates p < 0.05.

**Supplemental Table** 1: The 15 mutant strains whose growth was inhibited in the low rifampicin concentration.

**Supplemental Table 2**: MICs of *M. marinum* wild type and mutant strains.

**Supplemental Table 3:** MIC for CCCP of *M. marinum* wild type, *kdpA* mutant, and complemented strains.

**Supplemental Table 4**: List of primers used in this study.

Supplemental Figure 1

 Supplemental Figure 2

Supplemental Table 1

| Gene name | Gene Locus | Transposon insertion sites | Product |
| --- | --- | --- | --- |
| *arsC* | MMAR_2055 | +131nt | arsenic-transport integral membrane protein ArsC |
| *umaA* | MMAR_0794 | -11nt | mycolic acid synthase UmaA |
| *MMAR_4275* | MMAR_4275 | -80nt | PadR-like transcriptional regulatory protein |
| *MMAR_0777* | MMAR_0777 | +78nt | conserved secreted protein |
| *oppB* | MMAR_4136 | +581nt | oligopeptide-transport integral membrane protein ABC transporter OppB |
| *udgA* | MMAR_0603 | +505nt | UDP-glucose dehydrogenase UdgA |
| *MMAR_0496* | MMAR_0496 | +574nt | conserved transmembrane protein |
| *MMAR_0976* | MMAR_0976 | +231nt | conserved hypothetical protein |
| *MMAR_3984* | MMAR_3984 | +8nt | PPE family protein |
| *MMAR_0183* | MMAR_0183 | +742nt | PE-PGRS family protein |
| *mas* | MMAR_1767 | +1298nt | multifunctional mycocerosic acid synthase membrane-associated Mas |
| *ftsW* | MMAR_3194 | +652nt | FtsW-like protein FtsW |
| *MMAR_0806* | MMAR_0806 | +635nt | PE-PGRS family protein |
| *kdpA* | MMAR_0631 | +75nt | potassium-transporting ATPase a subunit, KdpA |
| *MMAR_0461* | MMAR_0461 | +346nt | conserved hypothetical transmembrane protein |

“+131nt” indicates that the transposon insertion site is 131 nt after the transcription start codon. “-11nt” indicates that the transposon insertion site is 11 nt before the transcription start codon.Supplemental Table 2

| Strains | MIC (μg/mL ) | | | | |
| --- | --- | --- | --- | --- | --- |
|  | RIF | STR | INH | CFX | GEN |
| W.T. | 0.25 | 2.5 | 0.25 | 1.25 | 12.5 |
| *arsC*::Tn | 0.25 | 2.5 | 0.25 | 1.25 | 12.5 |
| *umaA*::Tn | 0.25 | 2.5 | 0.25 | 1.25 | 12.5 |
| *MMAR_4275*::Tn | 0.25 | 2.5 | 0.25 | 1.25 | 12.5 |
| *MMAR_0776*::Tn | 0.25 | 2.5 | 0.25 | 1.25 | 12.5 |
| *oppB*::Tn | 0.25 | 2.5 | 0.25 | 1.25 | 12.5 |
| *udgA*::Tn | 0.25 | 2.5 | 0.25 | 1.25 | 12.5 |
| *MMAR_0496*::Tn | 0.25 | 2.5 | 0.25 | 1.25 | 12.5 |
| *MMAR_0976*::Tn | 0.25 | 2.5 | 0.25 | 1.25 | 12.5 |
| *MMAR_3984*::Tn | 0.25 | 2.5 | 0.25 | 1.25 | 12.5 |
| *MMAR_0183* | 0.25 | 2.5 | 0.25 | 1.25 | 12.5 |
| *mas*::Tn | 0.03125 | 2.5 | 0.25 | 0.3125 | 3.125 |
| *fstW*::Tn | 0.0625 | 2.5 | 0.25 | 0.625 | 12.5 |
| *MMAR_0806*::Tn | 0.25 | 2.5 | 0.25 | 1.25 | 12.5 |
| *kdpA*::Tn | 0.25 | 2.5 | 0.25 | 1.25 | 12.5 |
| *MMAR_0461*::Tn | 0.25 | 2.5 | 0.25 | 1.25 | 12.5 |

RIF, rifampin; STR, streptomycin; INH, isoniazid; CFX, ciprofloxacin; GEN, gentamicin.

Supplemental Table 3

| Strains | MIC of CCCP (mM) | | |
| --- | --- | --- | --- |
|  | 7 mM K^+^ | 70 mM K^+^ | 140 mM K^+^ |
| W.T. | 0.125 | 0.125 | 0.125 |
| *kdpA*::Tn | 0.375 | 0.1875 | 0.125 |
| *kdpA*::Tn-p_SMT3L_-*kdpA* | 0.125 | 0.125 | 0.125 |

Supplemental Table 4

| Gene | Primer sequence | Comments |
| --- | --- | --- |
| MycoMarT7 | CGGGGACTTATCAGCCAACCTG | MycoMar-specific primers |
| *sigA* | FW-CTCAAGCAGATCGGCAAGGT | For qRT-PCR |
|  | RV-CGGTCTGTCAACTCAGCCAT |  |
| *kdpA* | FW- TTCCAGTTGATGCAGGGCAA |  |
|  | RV- GTTCGTGTTGGTGACGAAGC |  |
| *kdpB* | FW- GAGATCGCGCTCAACATCCT |  |
|  | RV- GGCCTTTGAGTAGATCGCCA |  |
| *kdpC* | FW- CTACCCGCTGTTGGTCTGG |  |
|  | RV- ACAGGTTTCCCGTCGACTTC |  |
| *kdpD* | FW- CAGATAACCGGCATCGAGCA |  |
|  | RV- AACGCTTCTGGGGTGATGTC |  |
| *kdpE* | FW- GACATGTCCGGCATCGAAGT |  |
|  | RV- TGCACCTTGTCCGACGAATC |  |
